# Mechanisms of sorafenib-induced cardiotoxicity: ER stress induces upregulation of ATF3, leading to downregulation of NDUFS1 expression and mitochondrial dysfunction

**DOI:** 10.3389/fphar.2025.1593290

**Published:** 2025-08-13

**Authors:** Ming Yan, Cheng Peng, Xueyan Lang, Yilan Li, Yao Zhang

**Affiliations:** ^1^ Department of Cardiology, The Second Affiliated Hospital of Harbin Medical University, Harbin, China; ^2^ Key Laboratory of Myocardial Ischemia, Ministry of Education, Harbin Medical University, Harbin, China; ^3^ Department of Cardiology, Harbin First Hospital, Harbin, China

**Keywords:** sorafenib, cardiotoxicity, ATF3, NDUFS1, ER stress, mitochondrial dysfunction, cell apoptosis

## Abstract

**Background:**

Sorafenib, a widely used tyrosine kinase inhibitor (TKI), has been associated with cardiotoxic effects; however, the precise molecular basis of this toxicity remains incompletely characterized. This study examined sorafenib’s impact on cardiac cells, focusing on endoplasmic reticulum (ER) stress signaling, specifically the PERK-eIF2α-ATF4 pathway and its downstream network.

**Methods:**

To elucidate these mechanisms, we employed a comprehensive approach integrating *in vivo* rat models, H9C2 cell-based assays, transcriptomic and proteomic profiling, along with biochemical validation techniques.

**Results:**

Our study reveals that sorafenib compromises cardiac function by inducing ER stress in cardiomyocytes, which activates the PERK-eIF2α-ATF4 pathway, leading to mitochondrial damage and apoptosis. These outcomes were supported by Western blot analysis and microscopic imaging, and were significantly mitigated following treatment with the ER stress inhibitor GSK2606414. Transcriptome data highlighted activating transcription factor 3 (ATF3) as the most prominently induced gene post-treatment. Further enrichment analysis identified several related pathways, while RT-PCR and immunoblotting confirmed ATF3 upregulation in H9C2 cells. Proteomic screening revealed NDUFS1 as a potential downstream effector. Silencing ATF3 via siRNA partially restored mitochondrial function, suggesting a negative regulatory effect of ATF3 on NDUFS1 that contributes to sorafenib-induced mitochondrial impairment.

**Conclusion:**

Collectively, these results uncover a critical signaling cascade—PERK/eIF2α/ATF4/ATF3/NDUFS1—involved in sorafenib-mediated cardiotoxicity and point to ATF3 modulation as a promising target for preventing or reducing cardiac injury caused by this drug.

## Graphical Abstract

**Figure:**
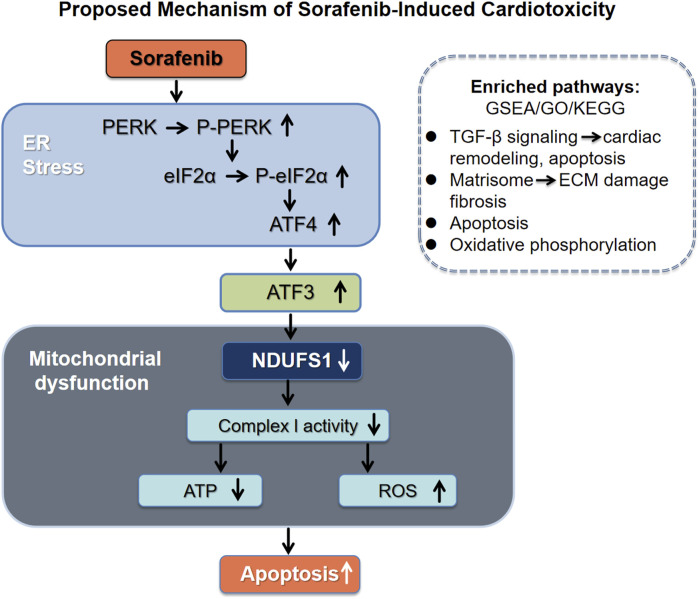
Schematic model illustrating the proposed mechanism of sorafenib-induced cardiotoxicity. Sorafenib triggers endoplasmic reticulum (ER) stress and activates the PERK-eIF2α-ATF4 signaling cascade, leading to upregulation of ATF3. ATF3 binds to the promoter of NDUFS1 and represses its expression, resulting in mitochondrial dysfunction, decreased mitochondrial complex I activity, reduced ATP production, elevated reactive oxygen species (ROS), and cardiomyocyte apoptosis. Transcriptomic and proteomic analyses also revealed enrichment of TGF-β signaling and Matrisome pathways, supporting the involvement of extracellular matrix remodeling and pro-apoptotic signaling in this process.

## 1 Introduction

Sorafenib is a first-line treatment for advanced hepatocellular carcinoma (HCC) (Chen et al., 2008; Fan et al., 2020; Zhou et al., 2022) and is approved for differentiated thyroid cancer (DTC) resistant to radioactive iodine therapy, as well as advanced renal cell carcinoma (RCC) (Escudier et al., 2019). Its anti-tumor efficacy extends to FLT-3 ITD-Mutant Acute Myeloid Leukemia, advanced desmoid tumors, and has shown promise in osteosarcoma trials (Gounder et al., 2018; Pollyea et al., 2021). However, it is associated with significant cardiac complications, including biomarker increases, electrocardiographic abnormalities, impaired contractility, and heart failure (Albarrán et al., 2022). Studies indicate a higher-than-expected incidence of left ventricular dysfunction (4%–8%) and a significant risk of decreased ejection fraction (Zamorano et al., 2016; Chaar et al., 2018). Recent reports have documented instances of unexpected cardiac arrest and myocardial dysfunction in individuals (Schmidinger et al., 2008; Escalante et al., 2016; Calistri et al., 2017). This underscores the need for a deeper understanding of sorafenib-induced cardiotoxicities.

The endoplasmic reticulum is vital for maintaining cellular homeostasis by regulating the synthesis and proper folding of intracellular proteins. Excessive ER stress activates the unfolded protein response (UPR) to restore balance, but prolonged stress can lead to cell death, contributing to cell injury and heart failure (Hou et al., 2021). The PERK/eIF2α/ATF4 pathway is a key element of the ER stress response and is closely linked to cardiac injury (Stevens et al., 2022). Studies show that arsenic and fluoride exposure induces ER stress, causing cardiomyocyte apoptosis in rat hearts and H9C2 cells, with increased GRP78 expression and PERK pathway activation compared to controls (Li M. et al., 2022). Previous research has shown that sorafenib induces apoptosis through ER stress pathways, particularly the PERK-eIF2α-ATF4 signaling cascade (Wang et al., 2022; Wang et al., 2023). However, the precise mechanisms of sorafenib-induced cardiotoxicity remain unclear, and the transition of apoptosis during prolonged treatment requires further investigation.

This study aims to fill the knowledge gap by exploring the link between ER stress and sorafenib-induced cardiotoxicity through comprehensive computational analysis and experimental validation. It focuses on identifying differentially expressed genes (DEGs) related to ER stress that may serve as diagnostic markers and therapeutic targets. By elucidating the molecular mechanisms, the research intends to enhance understanding of the pathways leading to sorafenib-induced cardiac damage, supporting the development of strategies to reduce these adverse effects.

## 2 Materials and methods

### 2.1 Animals and animal models

Six-week-old male C57BL/6 mice were individually housed (up to four per cage) in ventilated racks under controlled conditions: The mice were maintained at 21°C ± 1°C with a 12-h light/dark cycle and 30%–50% humidity, and were randomly assigned to two groups (n = 3). Sorafenib (HY-10201, MedChemExpress LLC, New Jersey, United States) was administered intraperitoneally to the treatment group at 30 mg/kg per day, while the control group received a vehicle solution of 10% DMSO, 40% PEG300, 5% Tween-80, and 45% saline. Myocardial function was assessed 2 weeks after the final injection, followed by humane euthanasia under isoflurane anesthesia for heart collection. All procedures complied with the ARRIVE guidelines.

### 2.2 Echocardiography

Two weeks post daily injections, echocardiographic evaluations were conducted on lightly anesthetized animals (1%–2% isoflurane) using a GE Vivid E9 ML6-15-D Linear Probe (United States). M-mode imaging assessed left ventricular fractional shortening (FS), ejection fraction (EF), and the thickness of the left ventricular posterior wall during diastole (LVPWd) and systole (LVPWs).

### 2.3 Histology staining

Heart tissues were rinsed with phosphate-buffered saline (PBS) and fixed in 4% paraformaldehyde (PFA) for 24 h. The tissues were then embedded in paraffin, sectioned into 2 μm slices, and stained with hematoxylin and eosin (HE) following standard procedures (Solarbio, G1121, China). Images were captured using a Leica^®^ DMi8 light microscope from Germany.

### 2.4 Culture and cell line maintenance

The H9C2 rat cardiomyocyte cell line (CRL-1446) was obtained from the American Type Culture Collection (ATCC, United States) and cultured in a CO_2_ incubator with 5% CO_2_ using Dulbecco’s Modified Eagle’s Medium (DMEM, GIBCO, United States) supplemented with 10% fetal bovine serum (FBS, GIBCO, United States). Cells were routinely passaged at approximately 80% confluence. Prior to experiments, the medium was replaced with DMEM containing 0.5% FBS for 12 h. Experimental treatments involved 5 μM sorafenib in dimethyl sulfoxide (DMSO) for 48 h, with 0.1 nM DMSO as the control. Sorafenib was also combined with 1 μM GSK2606414 (MedChemExpress, United States). All experiments were performed independently three times, each in triplicate.

### 2.5 Calcein AM/PI dual staining

Cell viability was assessed using the calcein AM/PI Double Staining Kit (E-CK-A354, Elabscience). Following PBS rinsing, cells were incubated with calcein AM and propidium iodide for 20 min. Fluorescence microscopy revealed green fluorescence from calcein AM (Ex/Em = 494nm/517 nm) and red fluorescence from PI (Ex/Em = 535nm/617 nm).

### 2.6 Cell viability assay

Cell viability was evaluated using the Cell Counting Kit-8 (CCK-8, BS350A, Biosharp, China). A 100 μL sample from each culture medium was added to a 96-well plate and incubated at 37°C. After 48 h of treatment, 10 µL of CCK-8 reagent was added to each well, and the absorbance at 450 nm was measured with a Tecan Infinite 200 PRO Microplate Reader (Tecan Group Ltd., Switzerland).

### 2.7 Western blotting

Cell lysates were obtained from cell lines using RIPA buffer with protease inhibitors (Beyotime, P0013B, China), and protein concentrations were determined via the BCA assay (Beyotime, P0012, China). Protein samples (10–20 µg) underwent separation by SDS-PAGE. Initially, electrophoresis was conducted at 70V for 30 min, then at 110V for 90 min (Sevenbio, Beijing, China). Proteins were subsequently transferred to a PVDF membrane at 300 mA for 70 min (Millipore, China). Membranes were blocked with 5% nonfat milk for 1 h at room temperature, followed by an overnight incubation with primary antibodies at 4°C. The study employed the following antibodies. Cleaved caspase-9 p35 (D315) at 1:1000 dilution (Immunoway, YC0014), P-PERK at 1:1000 (Wanleibio, WL05295), PERK at 1:1000 (Wanleibio, WL03378), EIF2S1 (P-eIF2α) at 1:2000 (Abcam, ab32157), eIF2α at 1:500 (Wanleibio, WL01909), GRP78/BiP at 1:1000 (Wanleibio, WL03157), ATF-4 at 1:500 (Wanleibio, WL02330), GAPDH at 1:10000 (Abcam, ab181602), ATF-3 at 1:1000 (Immunoway, YT0387), cleaved caspase-3 at 1:500 (Wanleibio, WL01992), and NDUFS1 at 1:The membrane was incubated with an HRP-conjugated secondary antibody following treatment with the primary antibody (Immunoway, YT3017). Protein bands were detected and quantified using an ECL system (Tanon 5100, China) and analyzed with ImageJ software (NIH v1.8.0).

### 2.8 Immunofluorescent staining

H9C2 cells were cultured on sterile coverslips in 24-well plates. After treatment, cells underwent three PBS washes and were fixed with 4% paraformaldehyde for 20 min. Permeabilization was achieved using 0.2% Triton X-100 for 15 min, followed by blocking with 10% goat serum in PBS for 1 h. Cells were then incubated overnight at 4°C with an anti-P-eIF2α antibody. After PBS washing, cells were incubated with a CY3-conjugated secondary antibody for 1 h at room temperature in the dark. Nuclei were stained with DAPI for 10 min at room temperature, protected from light. Fluorescent images were captured using an Olympus microscope (Japan).

### 2.9 Apoptosis analysis using flow cytometry

The apoptotic ratio of cardiomyocytes was evaluated using flow cytometry with an Annexin V-FITC apoptosis detection kit (Seven Biotech, SC123, China). Cells were collected, washed twice with cold PBS, resuspended in binding buffer, and incubated with 5 µL Annexin V and 10 µL PI for 15 min at room temperature in the dark. Data analysis was performed using FACSDiva (v6.1.3, BD Biosciences) and FlowJo V10 (TreeStar).

### 2.10 Detection of apoptosis by *in situ* fluorescence

Cells were cultured in a 6-well plate at 37°C. After 48 h of treatment, the medium was discarded, and the cells were washed twice with cold PBS. They were then incubated with 5 µL of Annexin V and 10 µL of PI at room temperature in the dark for 15 min.

### 2.11 RealTime-qPCR

Total RNA was extracted from cell lines using TRIzol reagent (Invitrogen, United States). cDNA synthesis was conducted with the Transcriptor First Strand cDNA Synthesis Kit (Roche, Germany) according to the manufacturer’s protocol. Quantitative PCR (qPCR) utilized SYBR Green I Master Mix (Roche, Germany) on a CFX96 Touch Real-Time PCR Detection System (Bio-Rad, United States). Ct values were automatically calculated and normalized to GAPDH, serving as the internal control. Gene expression levels were determined using the 2^−ΔΔCT^ method. Primer sequences are listed in Supplementary Table S1.

### 2.12 Transmission electron microscopy

Transmission electron microscopy (TEM) was employed to examine the ultrastructural morphology of mitochondria and ER in cell lines. Following treatment, cells were collected, washed three times with precooled sterile 1×PBS, and fixed in 2.5% glutaraldehyde in 0.1M phosphate buffer (pH 7.4) at 4°C for 24 h. TEM was then used to visualize and capture the mitochondrial and ER structures.

### 2.13 Examination of ER-mitochondria contact interactions

Post-treatment, cells were incubated with MitoTracker Green and ER-Tracker Red (Beyotime Institute of Biotechnology) at 37°C for 30 min, then analyzed via a Carl Zeiss confocal microscope.

### 2.14 ChIP-qPCR

Chromatin immunoprecipitation (ChIP) assays were conducted using a ChIP kit (#Bes5001, BersinBio, China) according to the manufacturer’s instructions. H9C2 cells underwent pretreatment, trypsinization, collection, and cross-linking with 1% formaldehyde to capture protein-DNA interactions. Glycine was used to terminate cross-linking, followed by a 10-min ice incubation and chromatin fragmentation via sonication. The fragmented chromatin was immunoprecipitated using an ATF3 antibody (ab207434, Abcam) to isolate protein-DNA complexes. After multiple washes, the immunoprecipitated DNA was eluted and subjected to qPCR analysis. The primers used for the ChIP assay are listed in Supplementary Table S2.

### 2.15 siRNA transfection

ATF3-targeting siRNAs were designed and synthesized by Gene Pharma (China), and transfected using Lipofectamine 3,000 (Invitrogen, United States) according to the manufacturer’s instructions. The siRNA sequences are listed in Supplementary Table S3.

### 2.16 Mitochondrial complex I activity assay

The Mitochondrial Complex I Activity Assay Kit (Solarbio) was utilized to evaluate mitochondrial complex I activity as per the manufacturer’s guidelines. Enzymatic activity was measured by isolating complex I from 5 × 10^6^ cells and observing NADH oxidation at 340 nm over 2 min.

### 2.17 ATP assays

Cells in the exponential growth phase were collected by centrifugation at 14,000 × g for 2 min at 4°C and rinsed with ice-cold PBS. Lysis was performed using the Enhanced ATP Assay Kit (S0027, Beyotime Biotechnology, Shanghai, China). ATP levels were quantified according to the manufacturer’s protocol, with concentrations calculated from a standard curve and reported as nmol/OD730.

### 2.18 JC-1 staining

The JC-1 Kit (Beyotime, C2006, China) was employed to evaluate mitochondrial membrane potential (ΔΨm) following the manufacturer’s guidelines. H9C2 cells were exposed to the JC-1 probe for 20 min at 37°C, with CCCP (10 μM) serving as a positive control. Mitochondrial depolarization was assessed by calculating the ratio of JC-1 aggregates to monomers. Imaging was conducted using a cell auto-imaging system (DMi8, Leica^®^, Germany), capturing five images per well.

### 2.19 Mitochondrial ROS assay

Mitochondrial reactive oxygen species (ROS) production was evaluated using MitoSOX Red (MCE, 1003197–00-9, China) following the manufacturer’s instructions. Cells were cultured in 6-well plates at 37°C under static conditions. After 48 h of treatment, the medium was removed, and 100 μL of 5 μM MitoSOX solution was added to each well. The plate was gently agitated for uniform distribution and incubated at room temperature for 30 min. Cells were then washed twice with PBS for 5 min each and subjected to fluorescence imaging using a microscope.

### 2.20 Intracellular ROS assay

Intracellular ROS levels were assessed with a commercial detection kit (Beyotime, S0033S, China). Post-treatment, cells were incubated with 10 μmol/L DCFH-DA at 37°C for 20 min and subsequently washed thoroughly. Fluorescence signals were imaged under a fluorescence microscope in a blinded manner.

### 2.21 Quantification of cellular superoxide production

Intracellular superoxide levels were measured using dihydroethidium (DHE, Beyotime Institute of Biotechnology). After 48 h of treatment, H9C2 cells were exposed to 5 μM DHE for 30 min at 37°C in a dark, humidified environment. The cells were then rinsed with PBS, and fluorescence was observed under a microscope by an investigator unaware of the group assignments.

### 2.22 Data acquisition

The GSE146096 dataset was sourced from the Gene Expression Omnibus (GEO) database, as detailed in Supplementary Table S4. The dataset, accessible at//www.ncbi.nlm.nih.gov/geo/, comprises transcriptomic data from human cardiac cells treated with various FDA-approved kinase inhibitors. Generated via RNA sequencing on the Illumina HiSeq 2,500 platform, the dataset was last updated on 13 October 2020. Human heart tissue-derived primary cardiomyocytes were divided into four experimental groups (A, B, D, and E), each exposed to sorafenib for 48 h at different concentrations. 2.5 μM in one subgroup of Line A, 0.5 μM in the remaining A subgroups, and 1 μM for all subgroups in Lines B, D, and E. Group A included four sorafenib-treated samples and 13 controls; Group B had four treated and eight controls; Group D comprised three treated and seven controls; and Group E contained three treated and 11 controls (Supplementary Table S5). Data on transcription factor binding sites (TFBS) for NDUFS1 was sourced from the GeneCards database (https://www.genecards.org/).

### 2.23 Bioinformatics analysis

The mRNA expression profiles were processed using R software (v4.2.1). Differential expression analysis was conducted with the ‘DESeq2’ package, calculating log2 fold change (logFC) values and adjusted P-values. The Benjamini–Hochberg method was applied to control false discovery rates. Genes with adjusted P-values below 0.05 and absolute logFC greater than 1.0 were considered significantly differentially expressed. Functional enrichment analysis, including gene ontology (GO), kyoto encyclopedia of genes and genomes (KEGG) and gene set enrichment analysis (GSEA), was performed using the ‘clusterProfiler’ package. Data visualization was performed using ‘ggplot2’ for differentially expressed gene distribution plots, principal component analysis (PCA), Venn diagrams, GSEA enrichment maps, GO term charts, and KEGG pathway maps, while ‘ComplexHeatmap’ was used for heatmap generation. Potential binding sites for ATF3 and NDUFS1 were identified via the JASPAR database.

### 2.24 Statistics

Results are expressed as mean ± standard error of the mean (SEM) from a minimum of three independent replicates. Statistical analyses and figure creation were performed using GraphPad Prism (version 8.0.2; GraphPad Software, La Jolla, CA, United States). An unpaired Student’s t-test was used to assess differences between two groups, with F-tests confirming variance homogeneity. A p-value of less than 0.05 was considered statistically significant.

## 3 Results

### 3.1 Apoptosis contributes to sorafenib-induced cardiotoxicity

**FIGURE 1:**
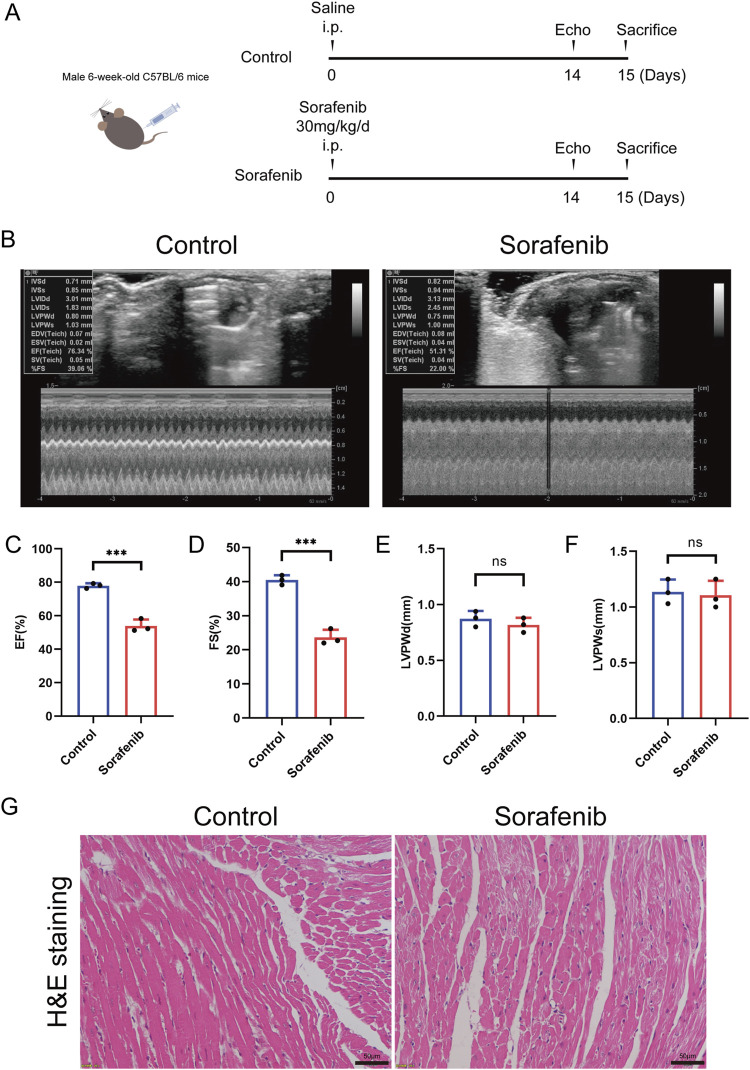
Sorafenib induces cardiac dysfunction in mice. **(A)** Diagram illustrates the comprehensive design framework. Mice received sorafenib at 30 mg/kg/day via intraperitoneal injection for 14 days. **(B)** Representative M-mode echocardiogram images from each study group. **(C)** EF, ejection fraction (n = 3 per group). **(D)** FS, fractional shortening (n = 3 per group). **(E,F)** LVPWd and LVPWs refer to the Left Ventricular Posterior Wall thickness measured at end diastole and end systole respectively (n = 3 per group). **(G)** Myocardial tissue damage was evaluated through hematoxylin and eosin (HE) staining, with a scale bar of 50 μm. Statistical significance was denoted as ns for non-significance, *P < 0.05, **P < 0.01, and ***P < 0.001 relative to the control group.

To establish a mouse model of sorafenib-induced cardiac injury, C57BL/6 mice were administered intraperitoneal injections of sorafenib following a dosing regimen that mimics clinical application (Figure 1A) (Wilhelm et al., 2004). The results demonstrated that sorafenib treatment impaired cardiac function, as shown by reduced left ventricular systolic performance (Figures 1B–F). Furthermore, hematoxylin-eosin (HE) staining provided histological evidence of myocardial injury. As shown in Figure 1G, sorafenib exposure led to myocardial fiber disarray and cardiomyocyte atrophy, along with cytoplasmic condensation, cell rounding, nuclear pyknosis, fragmentation, and dissolution.

**FIGURE 2:**
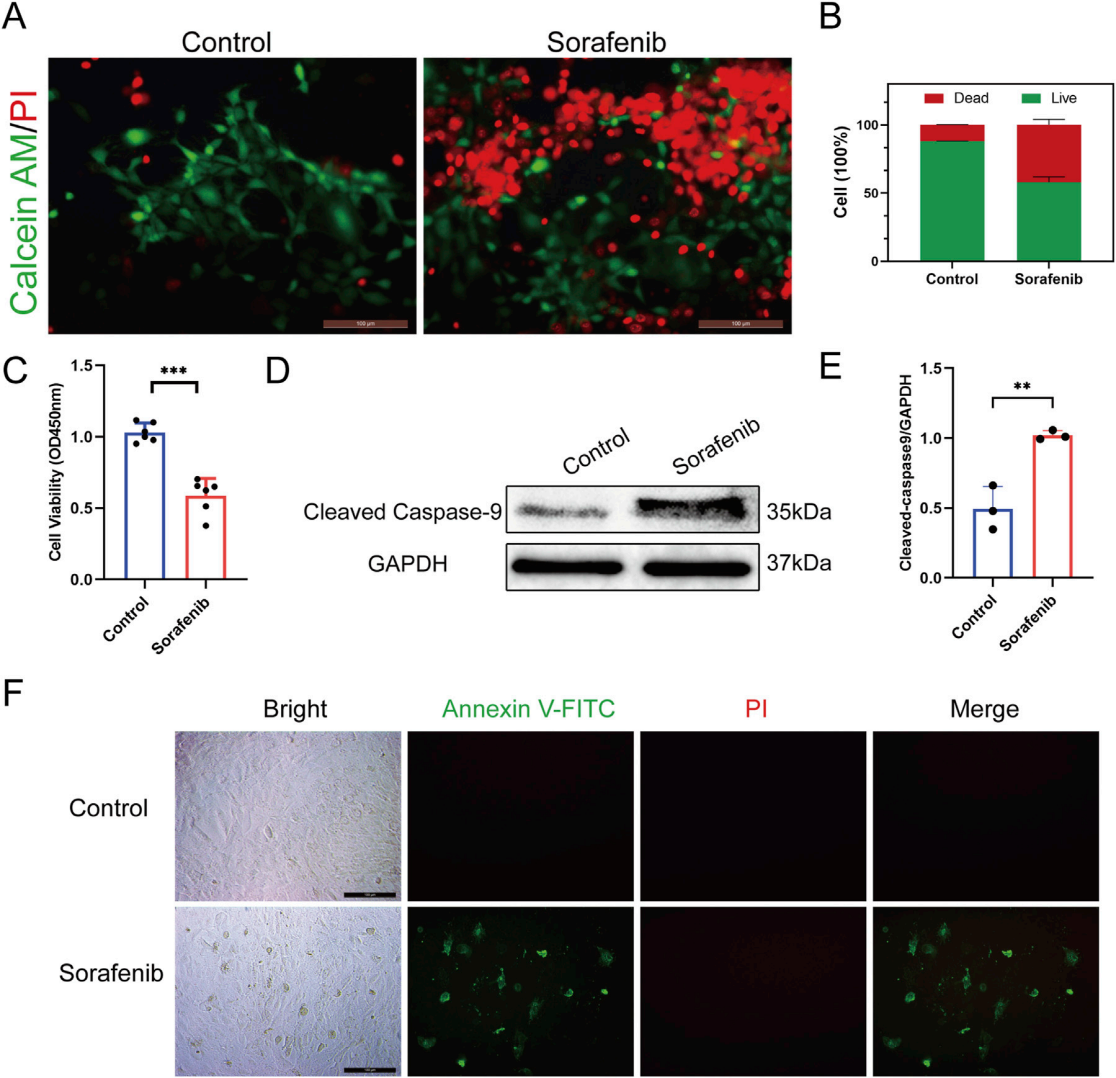
Sorafenib reduces viability in H9C2 cells and induces apoptosis. **(A,B)** Calcein AM/PI double staining was employed to evaluate sorafenib’s effect on H9C2 cell viability. Green: live cells (Calcein AM); red: dead cells (PI). n = 3 per group. Scale bar = 100 μm. **(C)** CCK-8 assays further assessed sorafenib’s impact on H9C2 cell viability (n = 6 per group). **(D,E)** Cleaved caspase-9 expression was analyzed using Western blot (n = 3 per group). **(F)** The apoptosis rate in rat cardiac H9c2 cells was assessed via fluorescence microscopy, with a scale bar of 100 μm. Statistical significance was denoted as ns for non-significance, *P < 0.05, **P < 0.01, and ***P < 0.001 relative to the control group.

Our study evaluated sorafenib’s effects on rat cardiomyocyte viability using calcein AM/PI dual staining and CCK-8 assays, revealing reduced cell viability compared to controls (Figures 2A–C). Western blot analysis showed increased cleaved caspase-9 expression in sorafenib-treated cells, indicating apoptosis contributes to sorafenib-induced cardiomyocyte toxicity (Figures 2D,E). Apoptosis in rat cardiac H9C2 cells was also assessed by *in situ* fluorescence after treatment with Annexin V and PI. Sorafenib treatment significantly increased apoptosis compared to controls (Figure 2F).

Apoptosis is crucial in sorafenib-induced cardiotoxicity. This is supported by decreased cell viability, upregulation of cleaved caspase-9, and *in situ* fluorescence detection. Typical apoptotic morphology, such as nuclear shrinkage and fragmentation, was also observed in cardiomyocytes. These findings were consistently observed in both *in vivo* mouse models and *in vitro* rat cardiomyocyte experiments.

### 3.2 Endoplasmic reticulum stress mediates sorafenib-induced cardiomyocyte death

The PERK-eIF2α-ATF4 pathway plays a crucial role in cellular responses to endoplasmic reticulum stress (ERS) and is strongly linked to cardiotoxicity. This study developed a cardiomyocyte model to examine sorafenib-induced ER stress and cardiotoxicity, using sorafenib alone or with the PERK inhibitor GSK2606414 to evaluate the pathway’s involvement.

**FIGURE 3:**
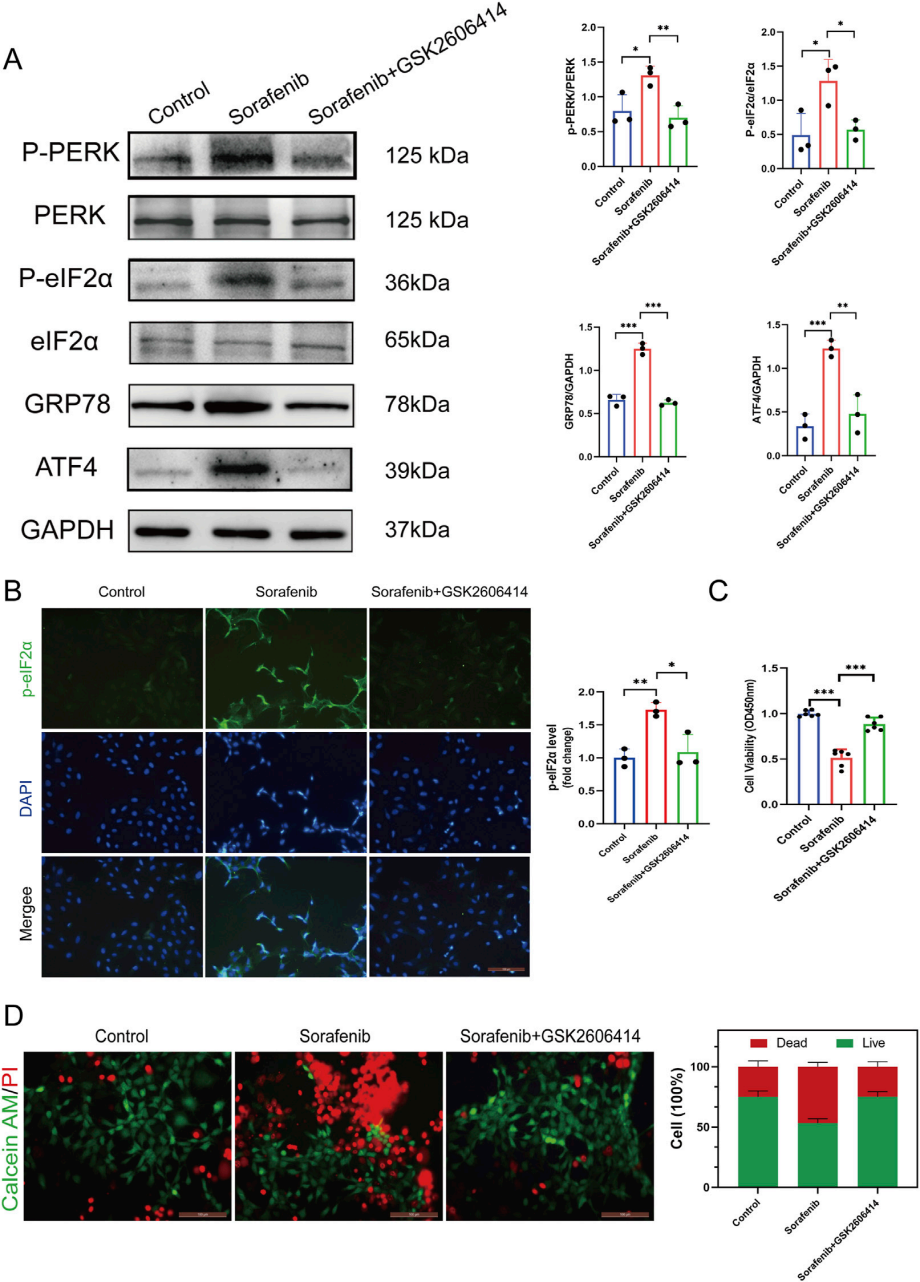
Endoplasmic reticulum stress inhibitor mitigates sorafenib-induced cardiomyocyte cell death. **(A)** Western blot analysis and quantification of P-PERK, PERK, P-eIF2α, eIF2α, GRP78, ATF4, and GAPDH. H9C2 cardiomyocytes were treated with 5 μM sorafenib (or vehicle) for 48 h. For rescue experiments, 1 μM GSK2606414 was co-administered. **(B)** Immunofluorescent staining was performed on cultured cells to analysis and quantification of P-eIF2α. **(C)** CCK-8 assays was performed to study how endoplasmic reticulum stress affects cell viability. **(D)** Calcein AM/PI staining assessed ER stress–induced changes in cell viability by distinguishing live and dead cells. Green: live cells (Calcein AM); red: dead cells (PI). Scale bar, 100 μm. Statistical significance was denoted as ns for non-significance, *P < 0.05, **P < 0.01, and ***P < 0.001 relative to the control group.

Western blotting and immunofluorescence staining were employed on cultured cardiomyocytes to investigate the PERK-eIF2α-ATF4 signaling pathway’s role in sorafenib-induced myocardial cell injury, as shown in Figures 3A,B. Sorafenib significantly increased PERK and eIF2α phosphorylation, evidenced by elevated p-PERK/PERK and p-eIF2α/eIF2α ratios, and upregulated ER stress markers GRP78 and ATF4. Pretreatment with the PERK inhibitor GSK2606414 reduced the pathway’s activation by sorafenib (Figures 3A,B), indicating its involvement in myocardial damage.

Furthermore, GSK2606414-mediated PERK inhibition mitigated sorafenib-induced cellular injury. To investigate endoplasmic reticulum stress (ERS) in sorafenib-induced cardiac injury, we performed the CCK-8 assay and calcein AM/PI double staining. Reduced cell viability (expressed as%) indicated a cardiomyocyte toxicity in the sorafenib-treated group *versus* the control group. Remarkably, the cardiotoxicity was attenuated by GSK2606414 treatment (Figures 3C,D). These findings suggest that inhibiting ERS mitigates sorafenib-induced cardiotoxicity in cardiomyocytes, highlighting the importance of the PERK-eIF2α-ATF4 signaling axis in myocardial cell injury.

### 3.3 Sorafenib induces cardiomyocyte apoptosis and subcellular alterations via endoplasmic reticulum stress

**FIGURE 4:**
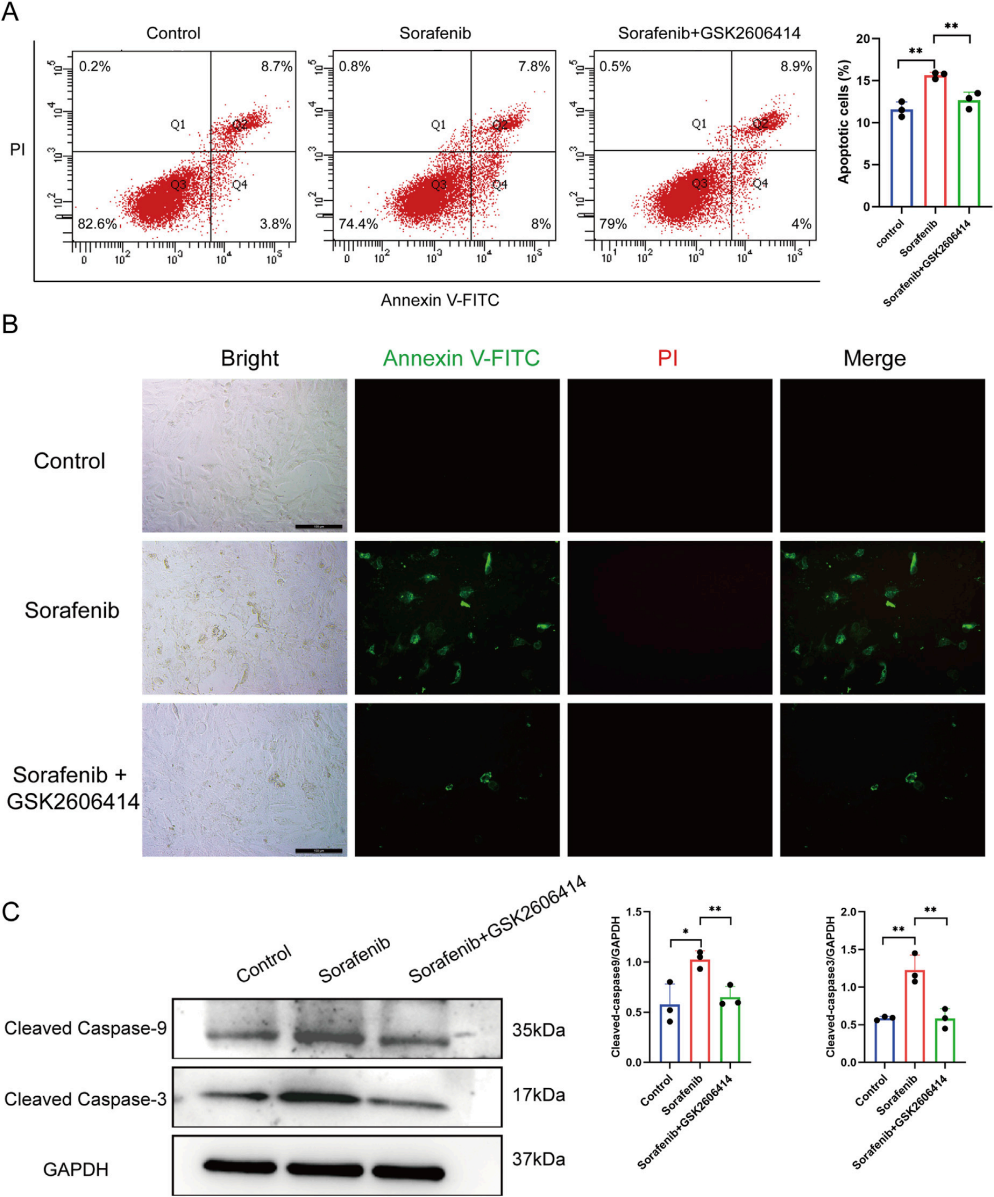
Inhibitors of endoplasmic reticulum stress can reduce cardiomyocyte apoptosis caused by sorafenib. **(A)** Cell apoptosis rate in rat cardiac H9c2 cells determined by flow cytometry. **(B)** The apoptosis rate in rat cardiac H9c2 cells was assessed via fluorescence microscopy, with a scale bar of 100 μm. **(C)** Western blot analysis was used to evaluate the expression of cleaved caspase-9 and cleaved caspase-3. Statistical significance was denoted as ns for non-significance, *P < 0.05, **P < 0.01, and ***P < 0.001 relative to the control group.

Apoptosis in rat cardiac H9C2 cells was assessed using flow cytometry and *in situ* fluorescence after treatment with Annexin V and PI. Sorafenib treatment significantly increased apoptosis compared to controls, an effect reduced by GSK26064 14 co-treatment (Figures 4A,B). Western blot analysis showed similar trends, with elevated levels of cleaved caspase-9 and cleaved caspase-3 in the sorafenib group, which were decreased by GSK2606414 (Figure 4C). The findings suggest that sorafenib induces cardiomyocyte apoptosis through ERS.

TEM analysis revealed mitochondrial damage in cardiomyocytes of the sorafenib-treated group compared to the control group, with significant variations in swelling and increased interaction with the ER. Notably, co-treatment with GSK2606414 mitigated the sorafenib-induced disruption (Supplementary Figure S1A).

Confocal imaging revealed that sorafenib treatment led to a marked increase in mitochondria–ER contact and co-localization, with obvious heterogeneity observed across the treated cells. This effect was notably suppressed by GSK2606414 (Supplementary Figure S1B). These findings suggest that sorafenib induces alterations in both the mitochondria and the ER. Notably, the application of GSK2606414 effectively delays these changes.

### 3.4 Characterization of differentially regulated genes involved in sorafenib-induced cardiotoxicity and functional enrichment analysis

**FIGURE 5:**
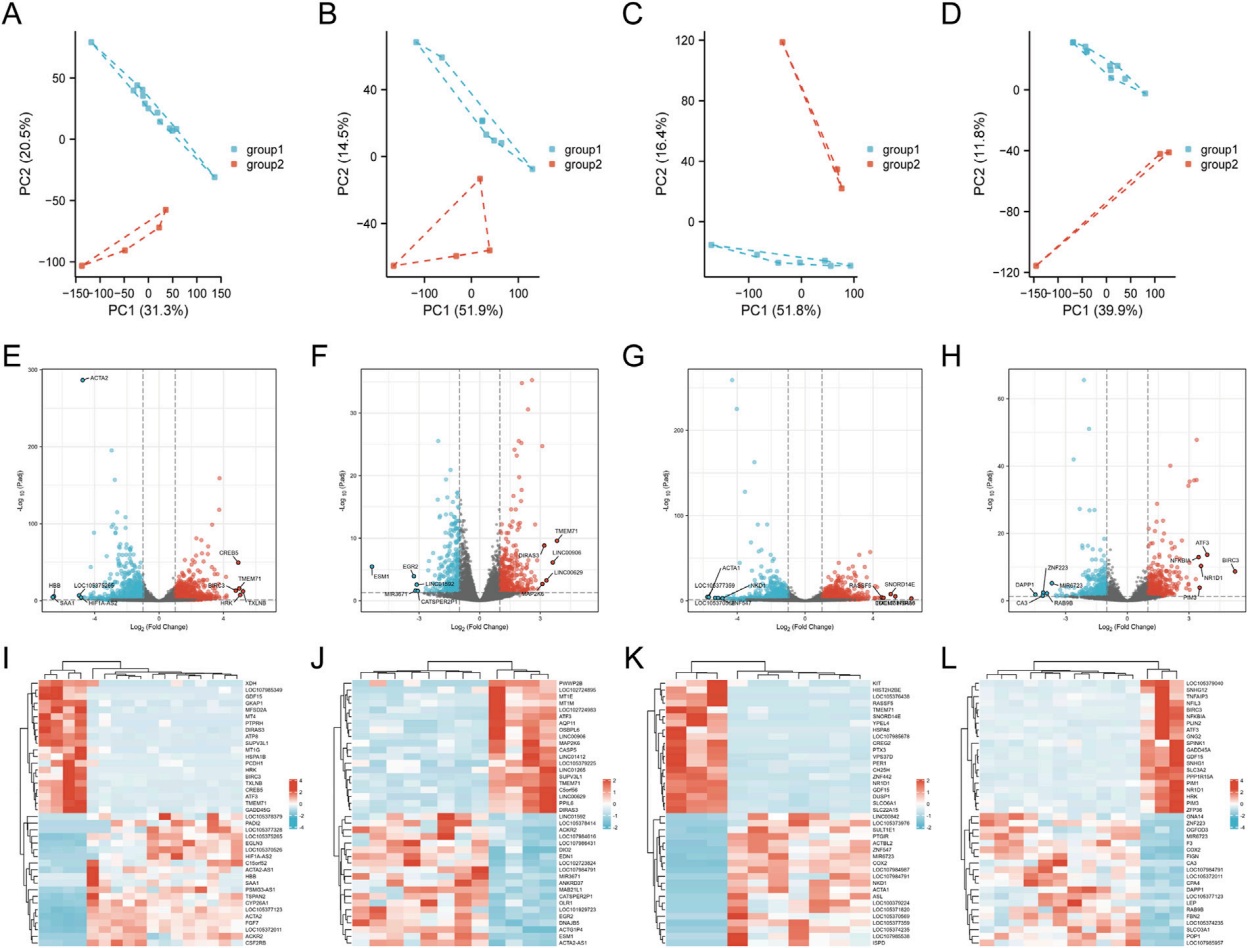
Identification of genes with differential expression linked to cardiotoxicity induced by sorafenib. **(A–D)** PCA score plots. **(E–H)** volcano plots of DEGs. **(I–L)** heatmaps for lines A, B, D, and **(E)**.

**FIGURE 6:**
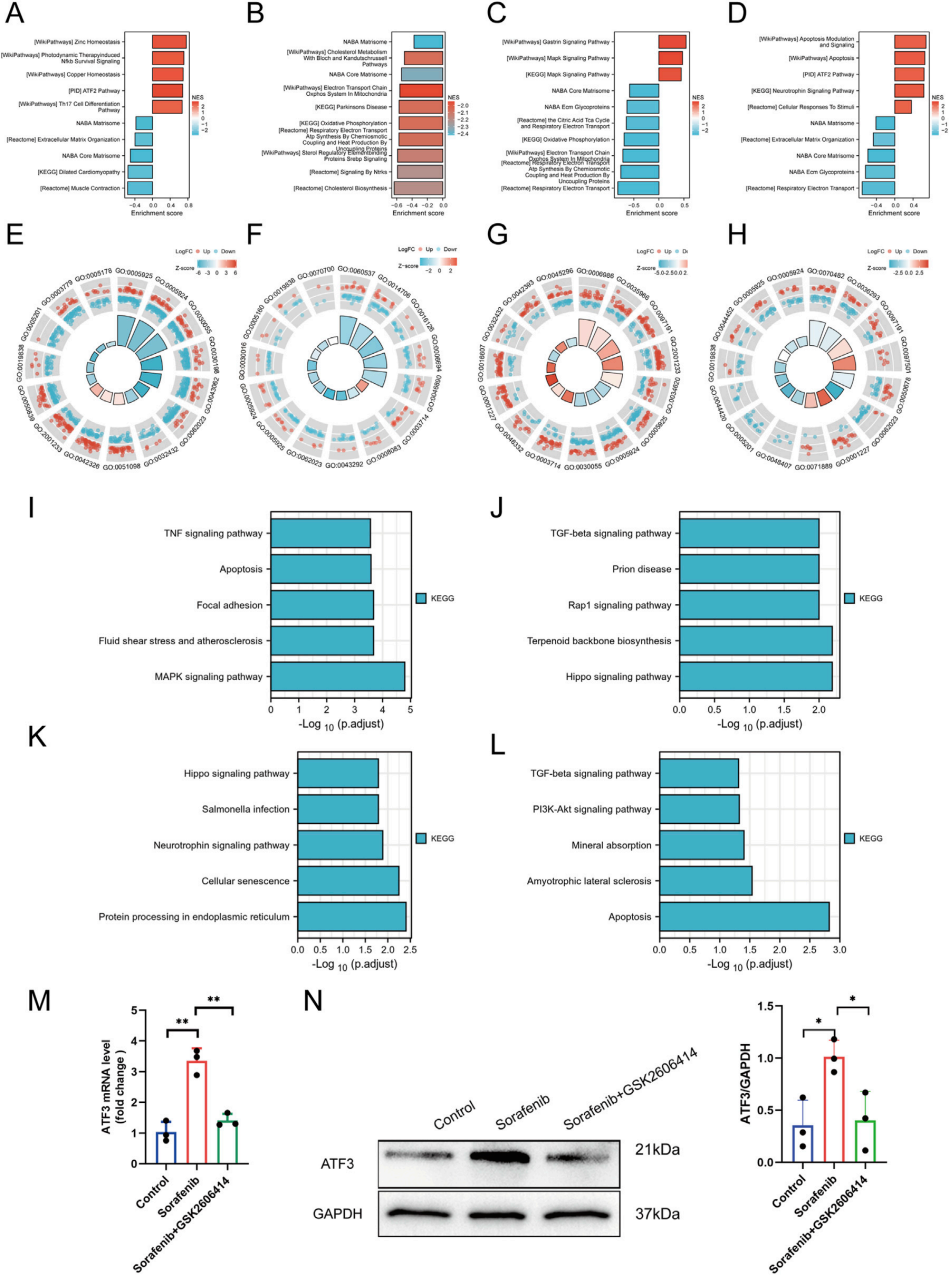
Functional enrichment analysis and experimental confirmation. **(A–D)** GSEA profiles for lines A, B, D, and **(E)**. **(E–H)** GO enrichment analyses for lines A, B, D, and **(E)**. **(I–L)** KEGG enrichment analyses for lines A, B, D, and **(E)**. **(M)** ATF3 mRNA expression detected by qRT-PCR. **(N)** ATF3 protein expression detected by Western blot. Statistical significance was denoted as ns for non-significance, *P < 0.05, **P < 0.01, and ***P < 0.001 relative to the control group.

Supplementary Figure S2 illustrates the analytical workflow. We examined mRNA expression data from the GSE146096 dataset, which includes four sorafenib-treated adult human cardiomyocyte samples, to investigate the transcriptomic response to sorafenib-induced cardiotoxicity. Data from the GEO database (Supplementary Table S4) was analyzed using PCA, revealing a distinct separation between the sorafenib-treated and control groups (Figures 5A–D), indicating a significant impact of the drug on the transcriptomic profile. DEGs were identified with thresholds of *P* < 0.05 and |logFC| > 1.0, and visualized through Volcano Plots and Heatmaps (Figures 5E–L). Further functional enrichment using GSEA, GO, and KEGG demonstrated significant clustering of DEGs. GSEA was performed on DEGs (Figures 6A–D), with detailed results in Supplementary Table S6, showing associations with pathways like NABA Matrisome, Oxidative Phosphorylation, and others. GO analysis identified key terms across biological process (BP), cellular component (CC), and molecular function (MF), including apoptotic signaling regulation, DNA-binding transcription factor interaction, and response to oxygen levels (Figures 6E–H; Supplementary Figure S3). KEGG analysis linked DEGs to pathways including apoptosis, TGF-β signaling, and protein processing in the ER (Figures 6I–L).

Among the significantly enriched pathways identified by GSEA, the TGF-β signaling pathway and Matrisome components were of particular interest due to their known relevance to cardiac pathology. TGF-β signaling plays a central role in cardiac fibrosis, hypertrophic remodeling, and pro-apoptotic responses under chronic stress conditions. Likewise, the Matrisome gene set, which includes structural extracellular matrix (ECM) proteins and remodeling enzymes, reflects early ECM disorganization—a hallmark of maladaptive cardiac remodeling. These changes may represent compensatory or maladaptive responses to mitochondrial dysfunction and ER stress induced by sorafenib.

### 3.5 Validation and functional characterization of differentially expressed genes as regulatory factors

A total of 77 upregulated and three downregulated cardiotoxicity-related DEGs were identified across line A, B, D, and E, as illustrated in the Venn diagrams (Supplementary Figure S4). These genes were then cross-referenced with 1,142 ERS-related genes obtained from the National Center for Biotechnology Information (www.ncbi.nlm.nih.gov.ncbi.nlm.nih.) (Supplementary Table S7). Fourteen differentially expressed ERS-related genes were identified, including ATF3, Growth Differentiation Factor 15 (GDF15), and protein phosphatase 1 (PP1), among others, as detailed in Supplementary Table S8. Among the differentially expressed ERS-related genes, the most significant differential expression was ATF3. Subsequently, to verify that sorafenib induces myocardial cell injury by activating ATF3 through ERS, Western blot and PCR were performed on cultured cells, and ATF3 was significantly upregulated (P < 0.05) (Figures 6M,N).

### 3.6 ATF3 interacts with the Ndufs1 promoter and suppresses its expression

**FIGURE 7:**
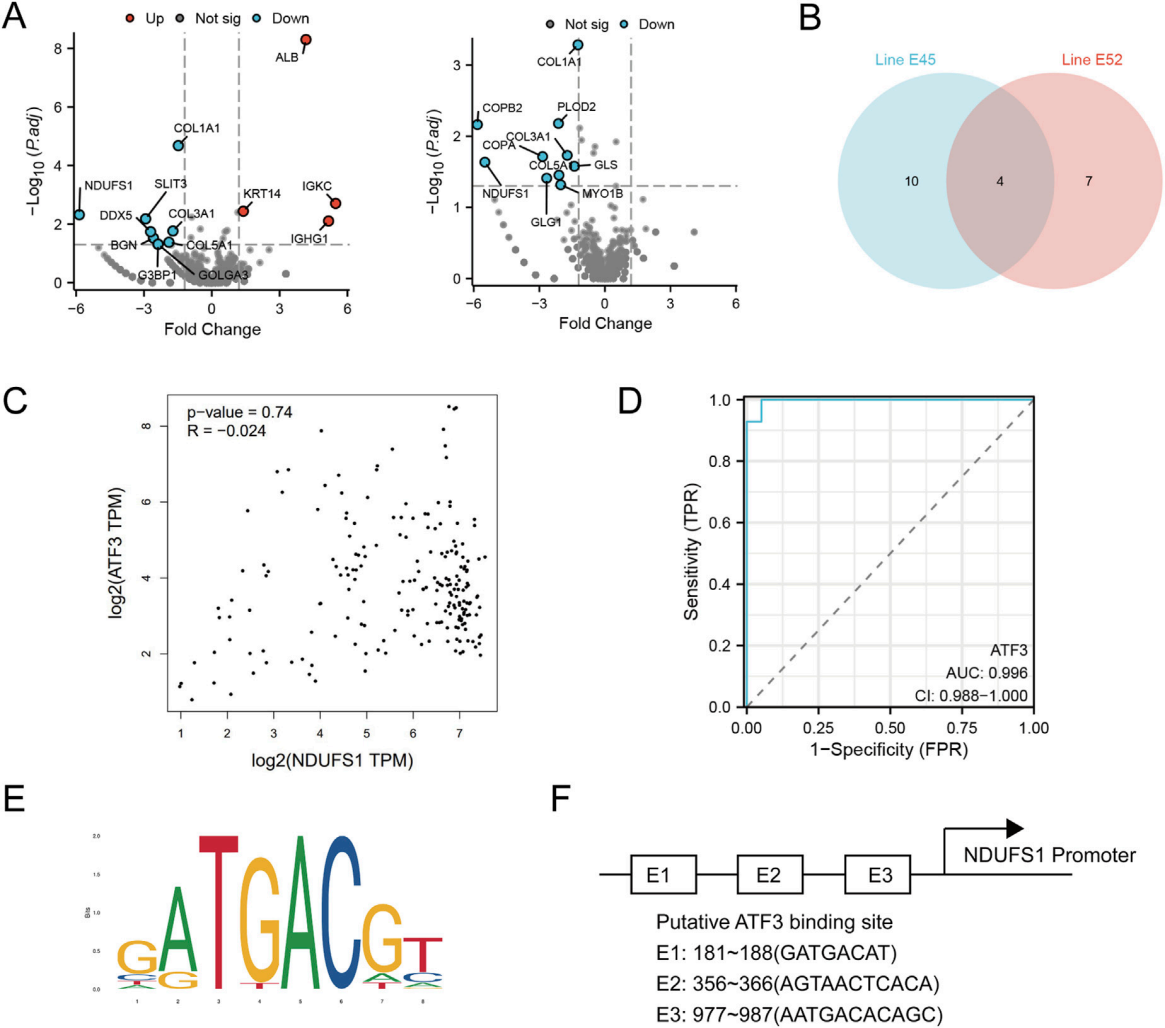
Proteomic analysis reveals ATF3 binds to the Ndufs1 promoter and negatively regulates its transcription. **(A,B)** Differentially expressed proteins in E45 and E52 were analyzed, with Venn diagrams showing their overlap. **(C)** Scatter plots from the GEPIA website, using GTEx database data, depict the correlation between Nudfs1 and ATF3 protein levels in H9C2 cells. **(D)** The regulatory sensitivity of ATF3 is evaluated. **(E,F)** The sequence logo of the NDUFS1 promoter region highlights ATF3 binding sites.

To understand the pathways involved in sorafenib-induced cardiotoxicity, we focused on ATF3’s role in cell survival and examined downstream proteins. Proteomic analysis of two sorafenib-treated cell lines identified four common differentially expressed proteins (DEPs), with NDUFS1 being the most significantly altered (Figures 7A,B). A Spearman correlation analysis revealed a negative relationship between ATF3 and NDUFS1 (Figure 7C). The regulatory sensitivity of ATF3 was evaluated using a ROC curve, yielding an AUC of 0.996, indicating excellent diagnostic performance (Figure 7D). Analysis of the NDUFS1 promoter region via the JASPAR database identified a strong ATF3 binding motif (Figures 7E,F).

**FIGURE 8:**
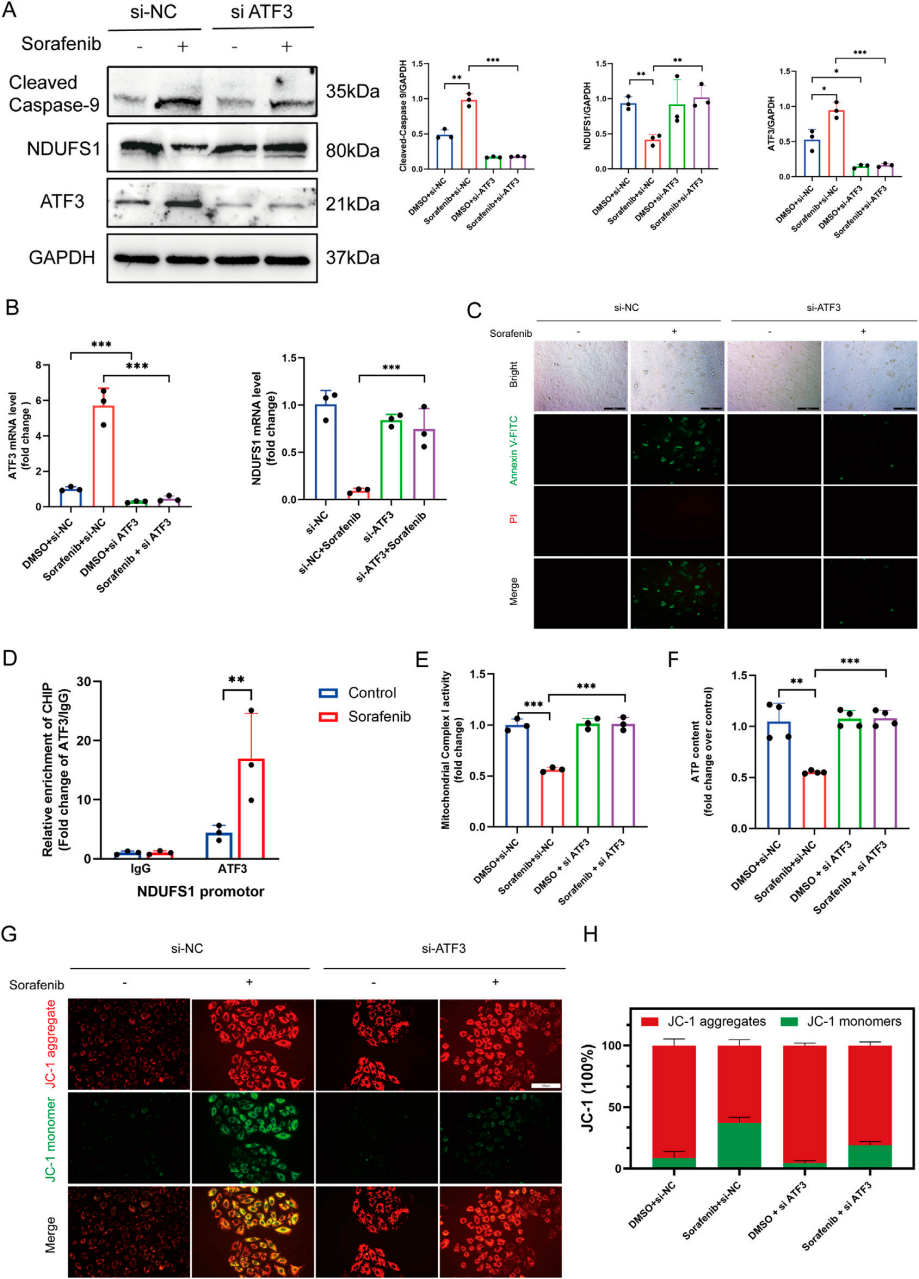
ATF3 binds to the Ndufs1 promoter suppressing its transcription and inducing apoptosis and mitochondrial dysfunction. **(A)** Western blot was used to assess the effect of ATF3 knockout on Ndufs1 and cleaved caspase-9 expressio (n = 3 per group). **(B)** RealTime-qPCR was used to assess the effect of ATF3 knockout on ATF3 and Ndufs1 (n = 3 per group). **(C)** The apoptosis rate in rat cardiac H9c2 cells was assessed via fluorescence microscopy, with a scale bar of 100 μm. **(D)** ChIP assays with an ATF3-specific antibody were conducted on H9C2 cells, and the precipitated DNA was quantified using real-time PCR (n = 3 per group). **(E)** Mitochondrial complex I activity was quantitatively analyzed in H9C2 cells (n = 4 per group). **(F)** ATP levels in H9C2 cells were quantified using an ATP Colorimetric Assay kit (n = 4 per group). **(G,H)** Mitochondrial membrane potential was evaluated via JC-1 staining. Statistical significance was denoted as ns for non-significance, *P < 0.05, **P < 0.01, and ***P < 0.001 relative to the control group.

Subsequent fundamental experiments validated the findings of the preceding proteomic analysis. ATF3 expression was notably lower in sorafenib-treated cells transfected with si-ATF3 compared to those with si-NC (negative control) (Figures 8A,B). Additionally, we demonstrated that sorafenib failed to downregulate NDUFS1 levels and enhance apoptosis-associated protein levels in ATF3-silenced cardiomyocytes (Figures 8A,B). *In situ* fluorescence staining revealed that ATF3 silencing did not significantly increase apoptosis in H9C2 cardiomyocytes treated with sorafenib (Figure 8C). Furthermore, based on the JASPAR database prediction, a chromatin immunoprecipitation-qPCR (ChIP-qPCR) analysis was performed, revealing enhanced ATF3 binding to the NDUFS1 promoter region following sorafenib treatment (Figure 8D; Supplementary Figure S5).

### 3.7 ATF3 knockdown reduces the susceptibility of H9C2 cells to sorafenib-induced mitochondrial impairment and oxidative damage

Cardiomyocyte function and viability are closely tied to mitochondrial energy production, with NDUFS1, a crucial subunit of mitochondrial complex I, playing a vital role in ATP synthesis and ROS generation. It is pivotal in regulating metabolic reprogramming, oxidative stress, and apoptosis in various diseases. Considering that ATF3-mediated downregulation of NDUFS1 aggravated sorafenib-induced cardiotoxicity, and TEM analysis revealed mitochondrial damage along with increased mitochondria-associated endoplasmic reticulum in sorafenib-treated H9C2 cells compared to controls (Figure 3A), we propose that sorafenib-induced cardiotoxicity may be linked to alterations in mitochondrial morphology and function. Mitochondrial complex I activity and ATP levels were significantly higher in the ATF3 knockdown group than in the sorafenib-treated group (Figures 8A,B). JC-1 staining indicated sorafenib-induced mitochondrial dysfunction in H9C2 cardiomyocytes, which was prevented by ATF3 knockdown (Figures 8C,D). Mitochondrial complex I is a major source of ROS. Compared to the sorafenib-treated group, ATF3 knockdown resulted in a significant reduction in mitochondrial ROS levels, as shown by MitoSOX staining (Supplementary Figure S6A,B). We assessed the impact of NDUFS1 depletion on oxidative stress by measuring ROS levels, finding a significant increase in ROS in sorafenib-treated cells, which was reduced in ATF3 knockdown cells, suggesting that ATF3 knockdown alleviates oxidative stress (Supplementary Figure S6C,D). Superoxide anion levels showed similar trends (Supplementary Figure S6E,F).

## 4 Discussion

Our study demonstrates that Sorafenib, a commonly used targeted therapy for cancers such as HCC and RCC, induces significant cardiotoxicity *in vivo* and *in vitro*. Our findings indicate that cardiotoxicity is associated with the activation of ER stress pathways, specifically the PERK-eIF2α-ATF4 cascade, which upregulates ATF3 and downregulates NDUFS1—a crucial mitochondrial complex I subunit (Bingchao et al., 2022)—thereby leading to mitochondrial dysfunction. The downregulation of NDUFS1 leads to reduced mitochondrial respiration, ATP depletion, increased oxidative stress, and ultimately results in cardiomyocyte apoptosis. Through our experiments, we found that silencing ATF3 with siRNA attenuates sorafenib-induced downregulation of NDUFS1 and significantly improves cardiomyocyte function. These results suggest that ATF3 is a key mediator of sorafenib-induced ER stress and mitochondrial dysfunction in cardiomyocytes. Therefore, our research offers novel mechanistic perspectives on the cardiotoxicity of sorafenib and underscores the significance of the ATF3-NDUFS1 axis in mediating sorafenib-induced cardiac injury.

### 4.1 Cardiotoxicity of sorafenib

Sorafenib has shown efficacy in treating various cancers, including HCC and RCC, but its use is limited by significant cardiotoxic side effects (Uraizee et al., 2011; Wu and Shemisa, 2017; Wang et al., 2023). Previous studies have demonstrated that sorafenib induces cardiovascular toxicity, primarily through mechanisms such as oxidative stress, myocardial apoptosis, endoplasmic reticulum stress and mitochondrial dysfunction (Ma et al., 2020; Bouitbir et al., 2022; Chen et al., 2023; Wang et al., 2023). However, there has been limited exploration of the specific molecular pathways involved, particularly those associated with endoplasmic reticulum stress and mitochondrial function regulation. Our findings address this gap by elucidating the involvement of ER stress signaling, specifically the ATF3-mediated pathway, in sorafenib-induced cardiotoxicity.

Although this study did not utilize a tumor-bearing model, previous reports have shown that sorafenib can induce cardiac dysfunction in both cancer patients and preclinical models independent of tumor burden (Jue et al., 2024). Investigating its cardiotoxic mechanisms in non-tumor models allows clearer assessment of drug-specific effects on cardiomyocytes, avoiding confounding factors associated with cancer biology. Nonetheless, future studies incorporating tumor models would help validate whether these mechanisms are preserved in more complex oncologic settings.

The *in vitro* concentration of 5 µM sorafenib used in this study was selected based on our group’s previous findings showing potent inhibitory effects on H9C2 cardiomyocytes (Li Y. et al., 2022; Yilan et al., 2024). While this dose may appear higher than physiological free drug levels, it remains below the reported total plasma C_max_ of 20–35 µM observed in patients receiving standard oral dosing (Dirk et al., 2004; Wilhelm et al., 2004). Given sorafenib’s high protein binding (>99.5%), the actual unbound fraction is significantly lower. Furthermore, sorafenib is known to accumulate in metabolically active tissues such as the heart, making localized exposure at or above 5 µM plausible. Therefore, this concentration is appropriate for modeling early cardiotoxic mechanisms under clinically relevant exposure conditions.

### 4.2 Endoplasmic reticulum stress and cardiotoxicity

ER stress, resulting from misfolded proteins, is associated with various diseases and can lead to apoptotic cell death if chronic or excessive (Almanza et al., 2019). It plays a significant role in heart failure and ischemic injury in cardiac tissues (Ren et al., 2021; Li M. et al., 2022; Preetha Rani et al., 2022). Our study builds on this by focusing on the activation crucial stress response proteins, in sorafenib-induced cardiotoxicity and its involvement in subsequent pathways. Specifically, our findings indicate that sorafenib activates the PERK-eIF2α-ATF4 signaling pathway in cardiomyocytes, leading to enhanced ER stress signaling and upregulation of stress-related markers. Pharmacological inhibition of PERK significantly attenuated this activation, suggesting that PERK functions as a key upstream mediator of sorafenib-induced ER stress. Furthermore, blocking this pathway alleviated cardiomyocyte injury, supporting the causal role of ER stress in sorafenib-induced cytotoxicity. These results not only reinforce the importance of ER stress in drug-induced cardiac damage but also identify the PERK-eIF2α-ATF4 axis as a potential therapeutic target for cardioprotection in patients receiving sorafenib.

Our omics data further supported this mechanistic link by revealing enrichment of TGF-β signaling and Matrisome-related pathways, both of which are intricately involved in cardiac stress remodeling and fibrosis. The activation of TGF-β signaling may represent a downstream consequence of ROS accumulation and apoptotic signaling triggered by ATF3-mediated mitochondrial dysfunction. Likewise, altered Matrisome gene expression may reflect early disruption of ECM homeostasis, which could sensitize cardiomyocytes to mechanical stress and cell death. These pathways may interact with the ATF3-NDUFS1 axis to amplify sorafenib-induced cardiac injury. Further studies are needed to dissect the temporal and causal relationship between these transcriptional signatures and the core mitochondrial damage axis.

### 4.3 The ATF3-NDUFS1 axis links ER stress to mitochondrial dysfunction and apoptosis in sorafenib-induced cardiotoxicity

Our study reveals a previously uncharacterized molecular axis by which sorafenib induces cardiotoxicity through ER stress and mitochondrial dysfunction. Sorafenib triggers the PERK-eIF2α-ATF4 signaling cascade, significantly increasing ATF3 expression, a stress-responsive transcription factor involved in apoptosis, inflammation, and metabolic regulation (Ge et al., 2023; Zhang et al., 2024; Gao et al., 2025). We demonstrate that ATF3 directly binds to the promoter of NDUFS1, a key component of mitochondrial complex I critical for electron transport and ATP synthesis (Bingchao et al., 2022), thereby suppressing its transcription.

The downregulation of NDUFS1 disrupts mitochondrial oxidative phosphorylation, reduces ATP synthesis, elevates ROS, and ultimately triggers cardiomyocyte apoptosis (Lothar and Judy, 2006; Irene et al., 2016; Zhuang et al., 2020; Kim et al., 2022). Notably, ATF3 knockdown restored NDUFS1 expression, improved mitochondrial membrane potential, reduced oxidative stress, and preserved mitochondrial bioenergetics. These results not only establish ATF3 as a critical modulator of mitochondrial function in drug-induced cardiac injury but also provide the first direct evidence linking the ATF3-NDUFS1 axis to sorafenib cardiotoxicity.

Previous studies have implicated ATF3 in stress-induced apoptosis across multiple tissues (Li et al., 2021; Yoo et al., 2021; Zhang et al., 2021; Gao et al., 2023), but its role in regulating mitochondrial function under pharmacologic stress was poorly understood. By integrating transcriptomics, proteomics, ChIP-qPCR, and functional assays, our findings position ATF3 as a dual effector—transducing ER stress signals and impairing mitochondrial function via NDUFS1 repression. Considering the pivotal function of mitochondria in cardiac physiology and the growing evidence linking mitochondrial dysfunction to cardiotoxicity (Jin et al., 2024; Junjun et al., 2025), this newly defined pathway offers both mechanistic insight and therapeutic targets. Targeting ATF3 or restoring NDUFS1 could serve as a viable approach to alleviate sorafenib-induced cardiac toxicity while preserving its anticancer efficacy.

Although this study demonstrated that ATF3 silencing alleviates mitochondrial dysfunction and restores NDUFS1 expression, further validation using ATF3 overexpression or NDUFS1 rescue approaches will be important to confirm the bidirectional regulatory relationship and to establish causality more robustly.

While our results demonstrate that sorafenib treatment and ATF3 upregulation are associated with marked repression of NDUFS1 and mitochondrial dysfunction, whether NDUFS1 downregulation alone is sufficient to recapitulate the full spectrum of mitochondrial impairment remains to be tested. We did not perform isolated NDUFS1 knockdown experiments in the absence of sorafenib in this study. Nevertheless, the tight temporal correlation between NDUFS1 suppression and key mitochondrial injury markers—including decreased Complex I activity, reduced ATP production, and ROS accumulation—strongly supports its central role. Future studies incorporating NDUFS1 loss-of-function models without sorafenib exposure will be essential to confirm whether NDUFS1 repression is sufficient, and not merely necessary, for driving the cardiotoxic phenotype.

### 4.4 Clinical and therapeutic implications of the ATF3–NDUFS1 axis in sorafenib-induced cardiotoxicity

The cardiotoxic effects of sorafenib pose a major limitation to its long-term clinical use, especially in patients without prior cardiovascular risk factors (Balachandran et al., 2024; Mohammed et al., 2024). Our findings reveal that the ATF3–NDUFS1 axis is not only a central mediator of mitochondrial dysfunction and cardiomyocyte apoptosis, but also a promising target for clinical intervention. The strong inverse correlation between ATF3 and NDUFS1, coupled with the ability of ATF3 knockdown to restore mitochondrial function and cell viability, suggests that these molecules may serve as sensitive biomarkers for predicting sorafenib-induced cardiotoxicity.

Therapeutic strategies aimed at inhibiting ATF3 or restoring NDUFS1 expression could potentially protect cardiac tissues from TKI-induced injury without compromising the anticancer efficacy of sorafenib. The development of selective ATF3 inhibitors, mitochondrial-protective agents, or gene therapy approaches may thus represent viable adjunct treatments to reduce cardiovascular side effects. Importantly, because ER stress is a shared mechanism across multiple chemotherapeutic and targeted agents, the ATF3-NDUFS1 axis may have broad applicability beyond sorafenib. Targeting this conserved stress-response pathway could form the basis for a generalizable cardio-oncology strategy aimed at preserving cardiac function during cancer therapy. Future research should confirm these results in clinical cohorts and evaluate the effectiveness of ATF3-targeted therapies in preclinical and human studies.

Although H9C2 cells provide a useful platform for mechanistic studies, their immortalized nature and embryonic rat origin may not fully represent the physiological responses of mature human cardiomyocytes. While our *in vivo* mouse model partially compensates for this limitation, future studies using more physiologically relevant systems—such as primary cardiomyocytes or human induced pluripotent stem cell–derived cardiomyocytes (hiPSC-CMs)—will be essential to validate the translational relevance of the ATF3–NDUFS1 signaling axis in sorafenib-induced cardiotoxicity. Nevertheless, H9C2 cells have been widely employed in early-phase cardiotoxicity research, particularly for studying endoplasmic reticulum stress, mitochondrial dysfunction, and apoptosis, due to their reproducibility and sensitivity to stress stimuli (Ma et al., 2020; Hou et al., 2021). These characteristics justify their use in this study to explore fundamental mechanisms before transitioning to higher-fidelity models.

In clinical settings, early detection of sorafenib-induced cardiac injury typically involves monitoring for symptoms such as fatigue, dyspnea, and chest discomfort, in addition to periodic evaluation of cardiac biomarkers (e.g., troponins, BNP/NT-proBNP) and imaging studies like echocardiography to assess left ventricular function. Electrocardiographic changes may also provide early warning signs. If cardiotoxicity is suspected, dose adjustment or temporary discontinuation of sorafenib is often recommended. Management may include supportive therapies such as β-blockers, ACE inhibitors, or mineralocorticoid receptor antagonists, which are commonly used in heart failure treatment. These measures can mitigate the progression of cardiac dysfunction while allowing continuation of anticancer therapy where feasible.

## 5 Conclusion

This study uncovers novel mechanisms regarding ER stress and the ATF3-NDUFS1 axis in Sorafenib-induced cardiotoxicity. By showing that ATF3 is essential in driving mitochondrial impairment and cell death in cardiomyocytes, we provide new targets for alleviating the cardiac side effects of Sorafenib. These findings also offer valuable insights for developing strategies to prevent or treat drug-induced cardiotoxicity, with potential applications for other cancer therapies. Exploring ATF3 inhibitors and mitochondrial protection strategies could lead to safer and more effective future cancer treatments.

## Data Availability

The datasets presented in this study can be found in online repositories. The names of the repository/repositories and accession number(s) can be found in the article/Supplementary Material.

## Supplementary material

SUPPLEMENTARY FIGURE S1 Structural changes of subcellular organelle caused by sorafenib. **(A)** Transmission electron microscope images are shown with the upper panel at a magnification corresponding to a 5 μm scale bar and the lower panel at a 2 μm scale bar. **(B)** Representative staining of ER tracker and MitoTracker (magnification: scale bar = 10 μm).

SUPPLEMENTARY FIGURE S2 Flow chart of the study design. The dataset GSE146096 was used to analyze differential gene expression between sorafenib-treated adult human cardiomyocytes and controls across four distinct groups. DEGs: differentially expressed genes; FC: fold change.

SUPPLEMENTARY FIGURE S3 GO and KEGG combined with logFC bar chart.

SUPPLEMENTARY FIGURE S4 Venn diagrams showing the number of overlapping DEGs among lines **(A–E) (A)** Upregulated DEGs. **(B)** Downregulated DEGs.

SUPPLEMENTARY FIGURE S5 The JASPAR database was utilized to predict ATF3 binding sites within the NDUFS1 promoter.

SUPPLEMENTARY FIGURE S6 Reducing ATF3 expression decreases the susceptibility of H9C2 cells to sorafenib-induced oxidative stress. **(A,B)** MitoSOX staining was used to assess mitochondrial ROS levels. **(C,D)** Intracellular ROS levels were measured using the fluorescent probe DCFH-DA. **(E,F)** Dihydroethidium (DHE) staining was performed to evaluate superoxide anion levels. Statistical significance was denoted as ns for non-significance, **P* < 0.05, ***P* < 0.01, and ****P* < 0.001 relative to the control group.
