# Supplementary figures and images for: Mechanisms of sorafenib-induced cardiotoxicity: ER stress induces upregulation of ATF3, leading to downregulation of NDUFS1 expression and mitochondrial dysfunction
Source: Front Pharmacol. 2025 Aug 13;16:1593290. doi: 10.3389/fphar.2025.1593290 (PMC12380775; doi:10.3389/fphar.2025.1593290)

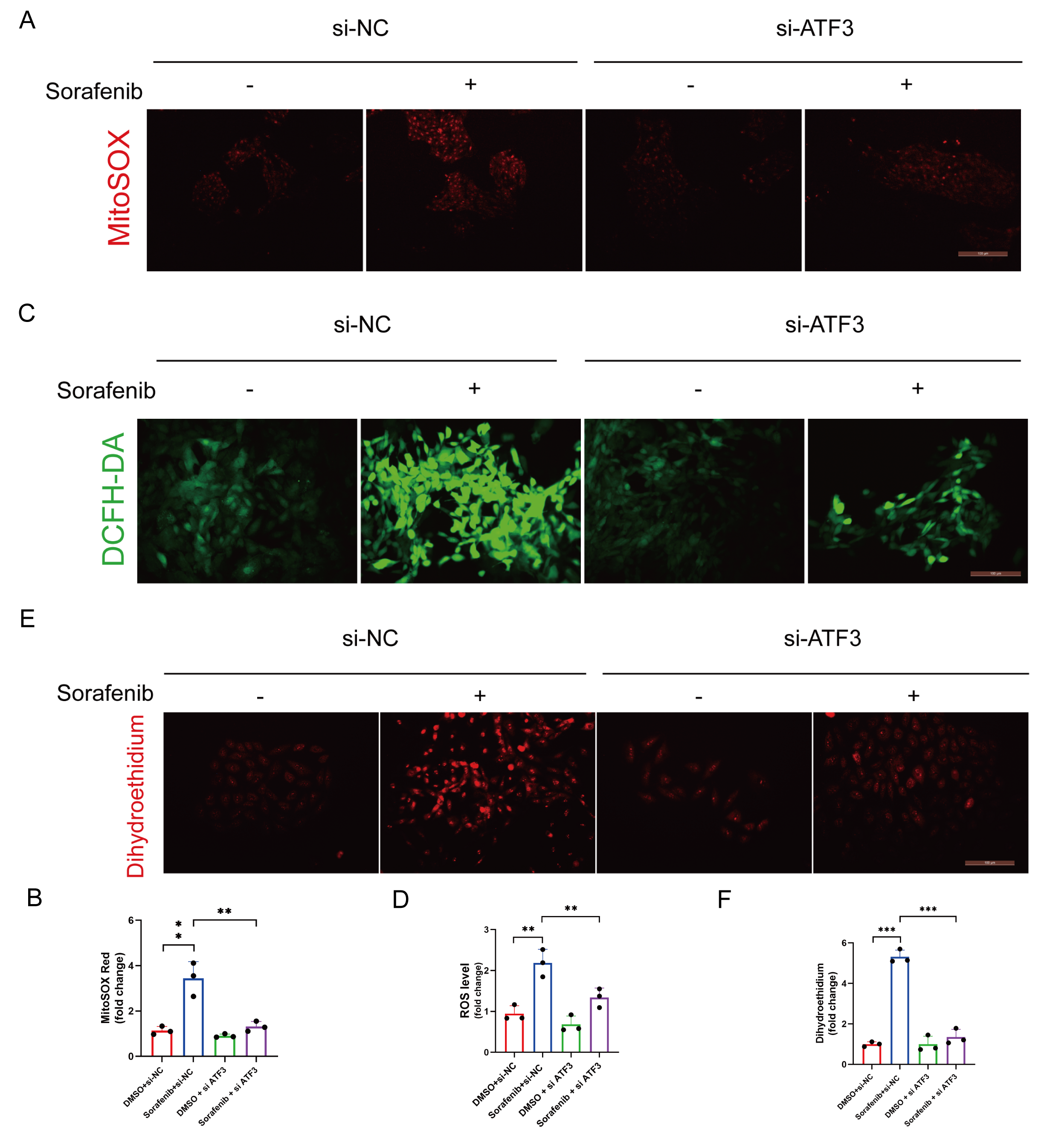

Supplement: Supplementary file 3 [file Image6.tif]

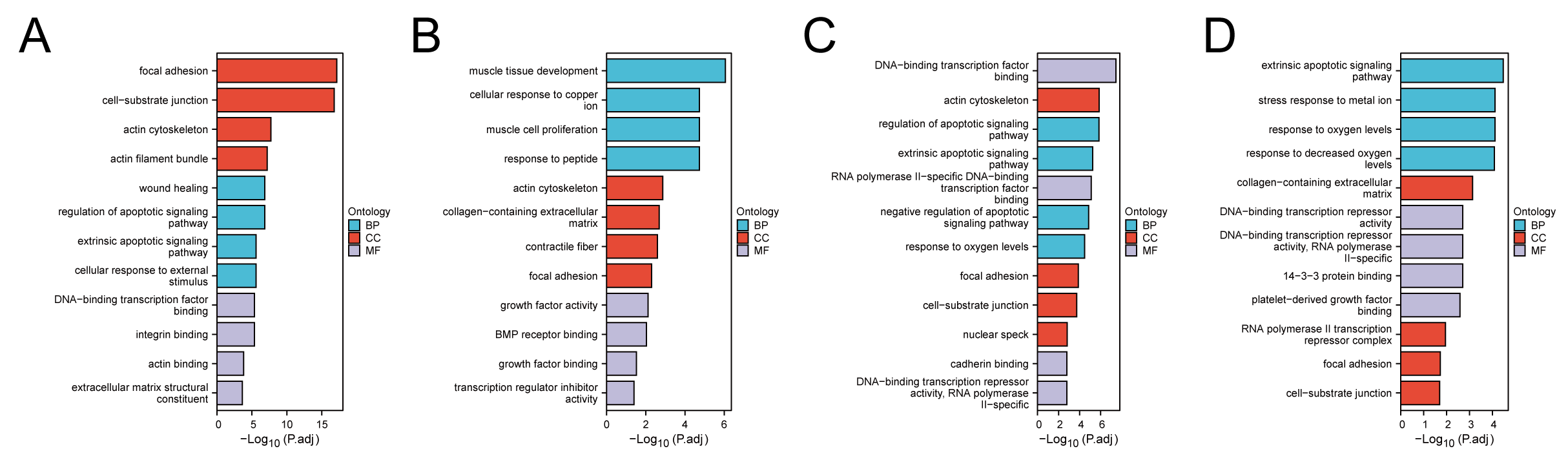

Supplement: Supplementary file 4 [file Image3.tif]

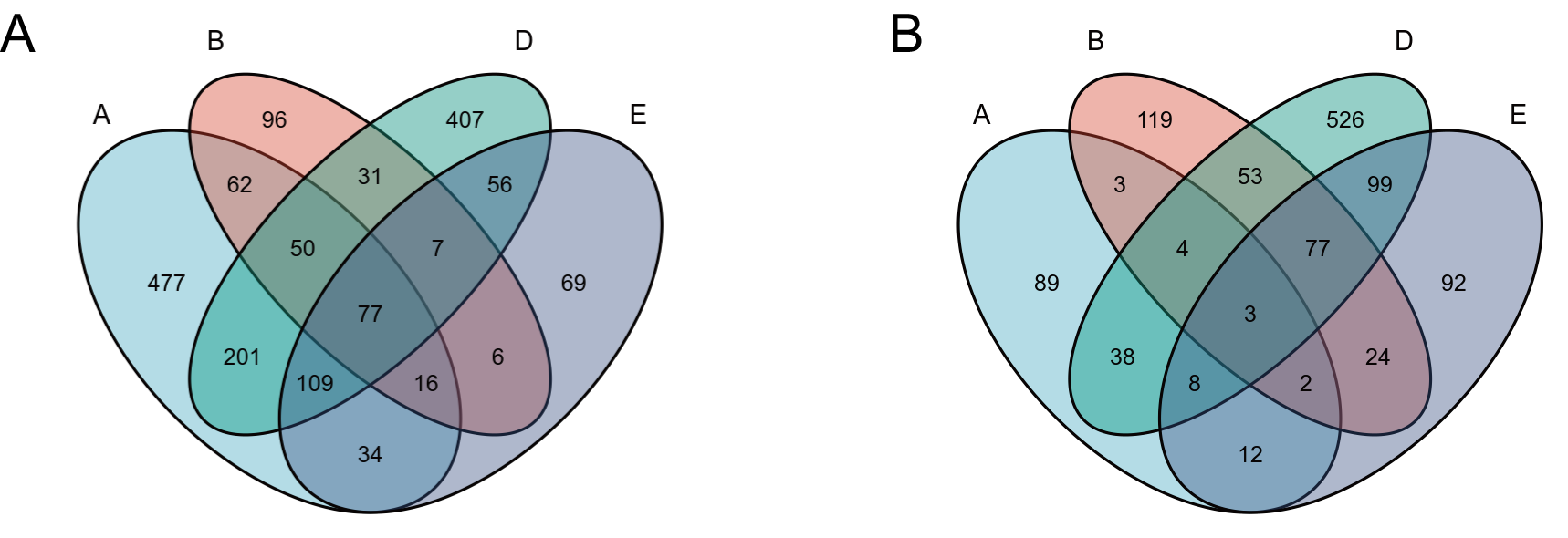

Supplement: Supplementary file 5 [file Image4.tif]

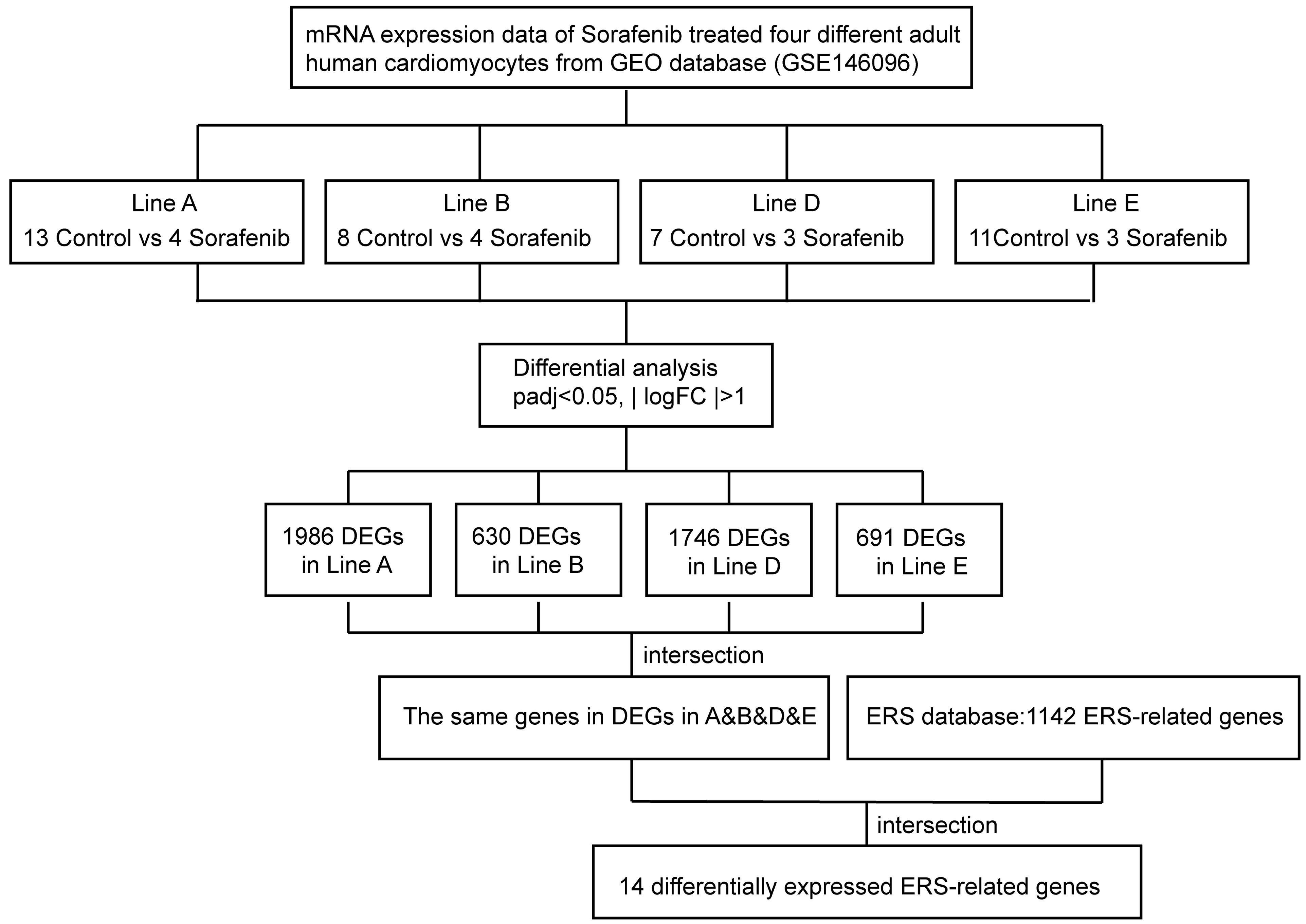

Supplement: Supplementary file 6 [file Image2.tif]

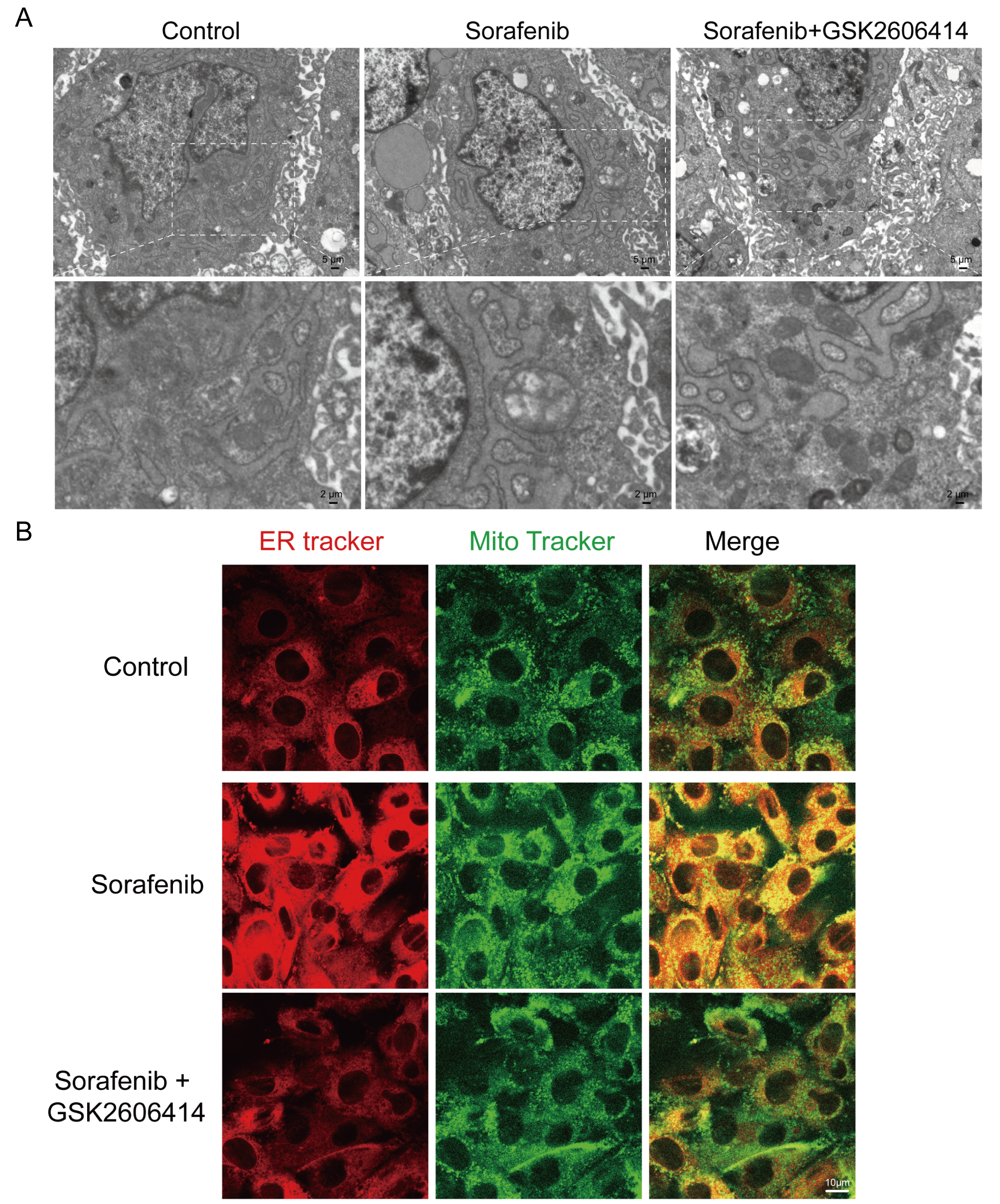

Supplement: Supplementary file 7 [file Image1.tif]

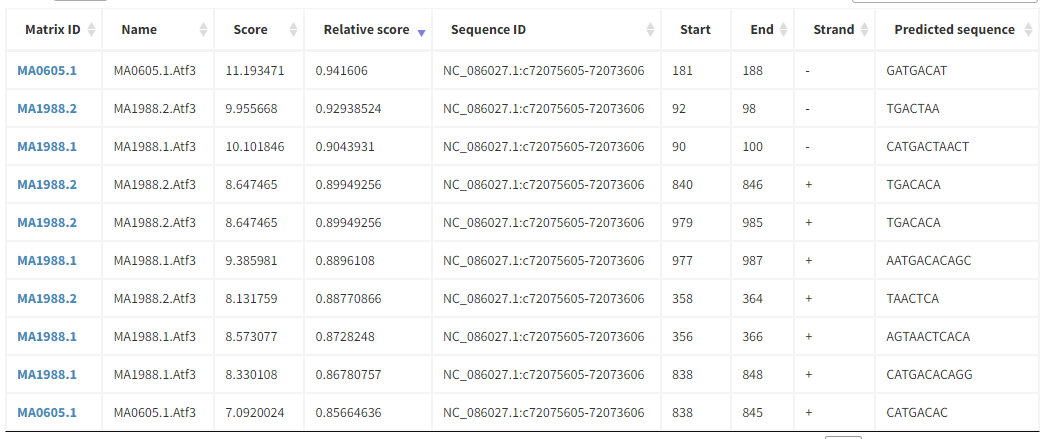

Supplement: Supplementary file 8 [file Image5.png]
